# Nasal cytological evidence of chronic inflammation in the olfactory cleft in post-viral olfactory dysfunction

**DOI:** 10.1007/s00405-025-09302-2

**Published:** 2025-03-26

**Authors:** Gianluca Velletrani, Denise Fiorelli, Beatrice Francavilla, Marzia Nuccetelli, Sergio Bernardini, Simonetta Masieri, Stefano Di Girolamo

**Affiliations:** 1 Department of Otorhinolaryngology, University of Rome “Tor Vergata”, Rome, 00133 Italy; 2 Department of Experimental Medicine, University of “Tor Vergata”, Rome, 00133 Italy; 3 Department of Oral and Maxillofacial Sciences, Sapienza University, Rome, 00185 Italy

**Keywords:** Olfactory dysfunction, Nasal cytology, Post-Viral, Lymphocytes, Ciliocytophthoria, Long COVID

## Abstract

**Purpose:**

This study investigated nasal cytological alterations in patients with persistent post-viral olfactory dysfunction. The primary objective was to evaluate the role of immune dysregulation and chronic local inflammation within the nasal mucosa in sustaining long-term olfactory impairment.

**Methods:**

An observational case-control study was conducted at the Otorhinolaryngology Department of the University of Rome Tor Vergata. Thirty-six patients with persistent olfactory dysfunction were compared to two control groups: one comprised subjects recovered from SARS-CoV-2 infection without olfactory impairment, and the other included individuals without a history of COVID-19 or olfactory dysfunction. Psychophysical olfactory function was assessed using the TDI (Threshold, Discrimination, and Identification) test. Nasal cytology samples were obtained via nasal brushing at the level of the olfactory cleft and stained using the May-Grunwald-Giemsa technique. Cellular alterations were evaluated using a semiquantitative grading system.

**Results:**

Patients with persistent olfactory dysfunction exhibited increased lymphocytes and neutrophils compared to both control groups, indicating ongoing local inflammation. Ciliocytophthoria was notably present in a significant portion of the olfactory dysfunction group, while absent or minimally present in controls. Eosinophils and mast cells were rare across all groups.

**Conclusion:**

Persistent post-viral olfactory dysfunction is associated with sustained immune activation and epithelial damage localized to the olfactory cleft. Elevated lymphocytes, neutrophils, and ciliocytophthoria emphasize the role of chronic inflammation in the pathogenesis of prolonged olfactory deficits. These findings highlight the potential utility of targeted therapies to modulate immune responses and promote olfactory recovery in affected patients.

## Introduction

Olfactory dysfunction (OD), characterized by the partial or complete loss of the sense of smell, is a common clinical symptom associated with various sinonasal and neurological conditions [1]. Anosmia, the complete loss of smell, and hyposmia, the partial reduction in the ability to smell, significantly affect quality of life, impacting taste perception, safety, and social interactions. The olfactory epithelium, located in the upper part of the nasal cavity, plays a crucial role in the detection of odors [2, 3]. This specialized tissue comprises olfactory sensory neurons, sustentacular cells, and basal cells that continuously regenerate to maintain olfactory function [4]. Disruptions in the integrity or function of these components, often due to viral infections, can lead to temporary or permanent olfactory dysfunction.

Nasal cytology has proven to be an invaluable diagnostic tool in the evaluation of various nasal pathologies, including allergic rhinitis, chronic rhinosinusitis, and viral infections [5–8]. By analyzing the cellular composition of nasal mucosa samples, nasal cytology allows for the identification of specific inflammatory patterns and cellular alterations that provide insights into underlying disease mechanisms. In the context of viral infections, including SARS-CoV-2, nasal cytology can reveal significant cytopathological changes such as ciliocytophthoria, neutrophilic infiltration, and lymphocytic hyperplasia, reflecting the body’s immune response to viral insult [9–12].

During the COVID-19 pandemic, olfactory dysfunction emerged as a hallmark symptom of acute SARS-CoV-2 infection [13]. Reports indicated that up to 50–60% of individuals with COVID-19 experienced some degree of smell loss, often occurring abruptly and early in the course of the disease [14]. This anosmia or hyposmia is thought to result primarily from the infection of sustentacular cells in the olfactory epithelium, which support the olfactory sensory neurons [15, 16]. Although the majority of COVID-19-related olfactory dysfunction cases are transient, resolving within weeks, a subset of patients report persistent olfactory deficits that extend beyond the acute phase of the infection [17, 18].

Persistent olfactory dysfunction after SARS-CoV-2 infection, a component of what is now recognized as long COVID or as post-acute sequelae of SARS-CoV-2 infection (PASC), that is characterized by a wide array of symptoms that persist for weeks to months after the resolution of the acute phase of the disease [19–21]. Among these symptoms, olfactory dysfunction is notably prevalent, causing ongoing distress and disability in affected individuals [22, 23]. Studies suggest that this persistent post-viral dysfunction is associated with sustained inflammation and immune responses in the olfactory epithelium, even after the viral particles are no longer detectable [24–26]. The pathophysiology of long COVID olfactory dysfunction remains poorly understood, but it is hypothesized that ongoing local immune activation and chronic inflammation play a critical role [26–28].

Despite its recognized utility in diagnosing various nasal diseases [7, 29], the application of nasal cytology to persistent olfactory dysfunction remains underexplored, particularly in the context of long COVID and other post-viral infections. While previous studies have predominantly focused on systemic immune responses and general symptoms of long COVID [30–33], localized processes within the nasal mucosa have received limited attention. Minimally invasive techniques like olfactory cleft brushing offer precise sampling of the olfactory mucosa, enabling detailed cytological assessment of immune responses and epithelial changes [34]. Addressing this gap is essential to understanding the mechanisms of post-viral olfactory dysfunction and developing targeted therapeutic interventions.

The aim of our study was to investigate the cytological alterations in the olfactory cleft of patients with persistent post-viral olfactory dysfunction. By focusing on cellular profiles obtained through olfactory cleft brushing, we sought to identify potential signs of ongoing inflammation or immune dysregulation contributing to prolonged olfactory deficits. This study aims to address the gap in the current understanding of long COVID by highlighting the importance of localized immune responses and offering new insights that may inform future diagnostic and therapeutic approaches for patients experiencing persistent post-viral olfactory dysfunction.

## Materials and methods

### Study design

An observational case-control study was conducted at the Otorhinolaryngology Department of the University of Rome Tor Vergata between April 2022 and October 2022. The study involved 36 patients experiencing persistent olfactory dysfunction attributed to long COVID, observed 3 to 18 months after the initial SARS-CoV-2 infection. These patients constituted the olfactory dysfunction group (OD). Two control groups were also established: the first control group (CG1) comprised 18 individuals who had recovered from SARS-CoV-2 infection without any olfactory impairment 3 to 18 months post-infection, and the second control group (CG2) included 18 participants who had never contracted SARS-CoV-2 and had no history of olfactory dysfunction. Participants in all three groups were matched by age and gender (mean age for OD group: 52 years; CG1: 46 years; CG2: 45 years) to ensure comparability (Table 1).

The sample size was determined to ensure adequate statistical power to detect significant differences between groups while accounting for the specific inclusion criteria and rarity of long COVID-related olfactory dysfunction.

All participants provided informed written consent, and the study protocol was approved by the Ethical Committee of Policlinico Tor Vergata.

**Table 1:** Demographic and clinical characteristics of study groups. Summary of demographic and clinical characteristics for the olfactory dysfunction (OD) group, control group 1 (CG1), and control group 2 (CG2). The table includes information on mean age and age range, gender ratio (M/F), history of SARS-CoV-2 infection, time since infection, presence of olfactory dysfunction, and the TDI score (Threshold, discrimination, and Identification) indicating olfactory function

	OD Group (*n* = 36)	CG1 Group (*n* = 18)	CG2 Group (*n* = 18)
Mean Age (years, range)	52 (24–72)	46 (23–68)	45 (23–70)
Gender (M/F Ratio)	1.25	1.4	1.4
History of SARS-CoV-2 Infection	Yes	Yes	No
Time Since Infection (months)	3–18	3–18	N/A
Olfactory Dysfunction Present	Yes	No	No
TDI Score (mean, range)	17.5 (7–26)	38 (35–43)	37.2 (31–48)

### Clinical evaluation

Participants underwent a comprehensive clinical evaluation to exclude signs of chronic rhinitis or chronic rhinosinusitis. This evaluation included nasal endoscopy performed with a rigid video endoscope (Karl Storz GmbH, Tuttlingen, Germany) equipped with 30° and 0° angles, 15 cm length, and 3.0 mm diameter. The Lund-Kennedy scoring system [35] was used to systematically assess the presence of mucosal edema, hyperemia, crusting, and scarring. Nasal polyposis and significant nasal discharge were specifically excluded during endoscopic examination. In addition to endoscopic findings, patients’ clinical history and symptoms were carefully reviewed to identify any indications of chronic nasal conditions, including a history or signs suggestive of allergic rhinitis. Participants with such conditions were excluded from the study. Furthermore, all participants were instructed to discontinue any treatment with local corticosteroids, systemic corticosteroids, or antihistamines for a minimum of two weeks prior to the cytological sampling, ensuring that these treatments did not interfere with the results.

The olfactory dysfunction group was defined based on psychophysical assessment [36] and subjective clinical presentation. All patients in this group presented to medical attention with subjective complaints of hyposmia or anosmia directly and temporally related to a prior viral event. Only patients reporting a Visual Analog Scale (VAS) score of 4 or higher (indicating moderate-to-severe olfactory dysfunction) were included.

Olfactory function was evaluated psychophysically using the extended Sniffin’ Sticks test n-Butanol, provided by the Sniffin’ Test kit (Burghart Medizintechnik GmbH, Wedel, Germany). This test, standardized across Europe, includes three subtests: odor threshold, odor discrimination, and odor identification [37].

Odor threshold testing involved 16 triplets of n-butanol at varying dilutions, identifying the lowest concentration detectable across two consecutive trials. The odor discrimination subtest required participants to identify the unique odor in 16 sets of three pens, while the odor identification subtest involved recognizing 16 commonly encountered odors from four multiple-choice options. To prevent olfactory desensitization, a 30-second interval was maintained between each odor exposure. All tests were performed in a quiet, well-ventilated room.

The results of these subtests were combined into a composite TDI score (Threshold, Discrimination, and Identification). Participants were categorized as normosmic (TDI ≥ 31), hyposmic (TDI between 17 and 31), or anosmic (TDI < 17), with only those in the hyposmic or anosmic range included in the olfactory dysfunction group.

### Nasal cytology

Nasal cytology samples were collected using a minimally invasive brushing technique targeting the olfactory cleft, as described in a prior publication [34]. A cytology brush (GIMA Brush, GIMA, Gessate, Italy) was inserted under endoscopic guidance into the olfactory cleft and rotated three times to obtain cellular samples. The technique ensured specific sampling of the olfactory mucosa, avoiding contamination from adjacent nasal areas. No anesthetic was required as the procedure was painless, but it was essential that the operator was well-trained to ensure proper sampling.

Nasal cytology was performed using Gelardi’s technique, approved by the Italian Academy of Nasal Cytology (AICNA) [38].

Samples were immediately smeared on a glass slides, air-dried, and stained using the May–Grunwald–Giemsa (MGG) technique. Each slide was washed in tap water, air-dried, and mounted in synthetic resin before applying a cover glass. Microscopic examination was conducted using a light microscope (EUROStar III Plus, EUROIMMUN, Lübeck, Germany) equipped with 100x, 400x, and 1000x objective lenses, with oil immersion at 1000x.

At least 50 fields were examined at 1000x magnification to ensure an adequate cellular count. Cellular analysis was performed manually by two independent observers to reduce bias and ensure reproducibility. Semi-quantitative analysis was performed, with cellular grading on a scale of 0, 1+, 2+, 3+, and 4 + for each cell type. This grading system was originally suggested by Meltzer [39, 40] and later refined by Gelardi [38]. For lymphocytes, reference values are less frequently reported in the literature. Therefore, we followed the guidelines set by the Italian Academy of Nasal Cytology (AICNA) and by Gelardi [6] for rhinocytogram analysis, which recommend using the same grading scale for lymphocytes as that used for mast cells and eosinophils.

### Statistical analysis

Statistical analysis was performed using GraphPad Prism Software 9.1.1 (GraphPad Software, San Diego, California, USA). Due to the non-normal distribution of the data, the non-parametric Mann-Whitney test was employed. Results were expressed as median and interquartile range values. A p-value of less than 0.05 was considered statistically significant.

## Results

**Fig. 1:**
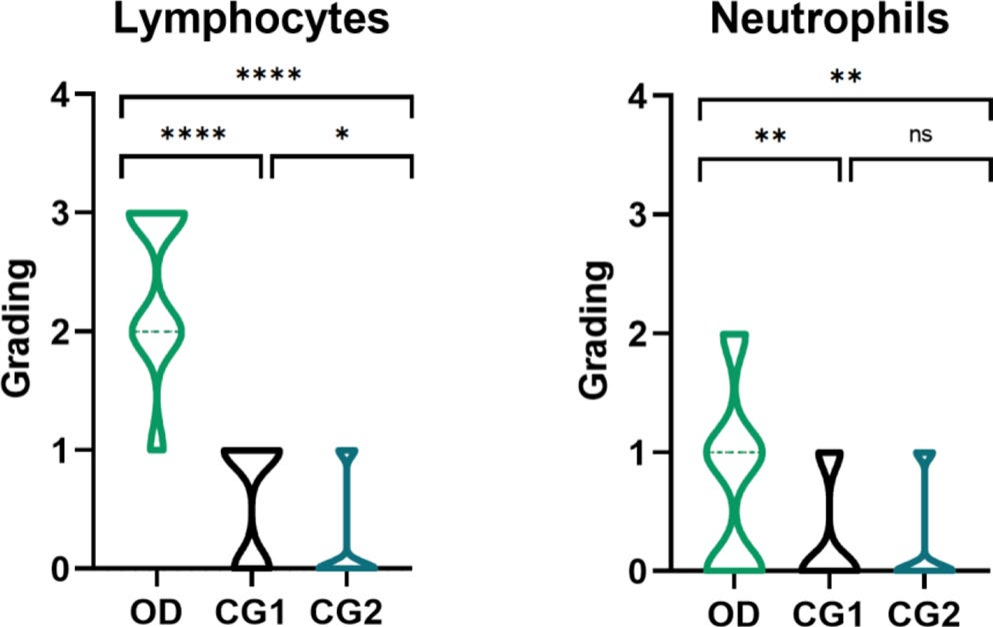
Comparison of Nasal Cytology Grading for Lymphocytes and Neutrophils Across Study Groups Violin plots illustrating the grading distribution of lymphocytes and neutrophils across the olfactory dysfunction (OD), control group 1 (CG1), and control group 2 (CG2) study groups. The y-axis represents the grading scale used in the semiquantitative nasal cytology analysis. For lymphocytes, the OD group shows a significantly higher grading compared to both CG1 and CG2, as indicated by **** (*p* < 0.0001) and * (*p* < 0.05). For neutrophils, the OD group also demonstrates significantly higher grading than both CG1 and CG2, with ** (*p* < 0.01), while no significant difference (ns) is observed between CG1 and CG2

**Fig. 2:**
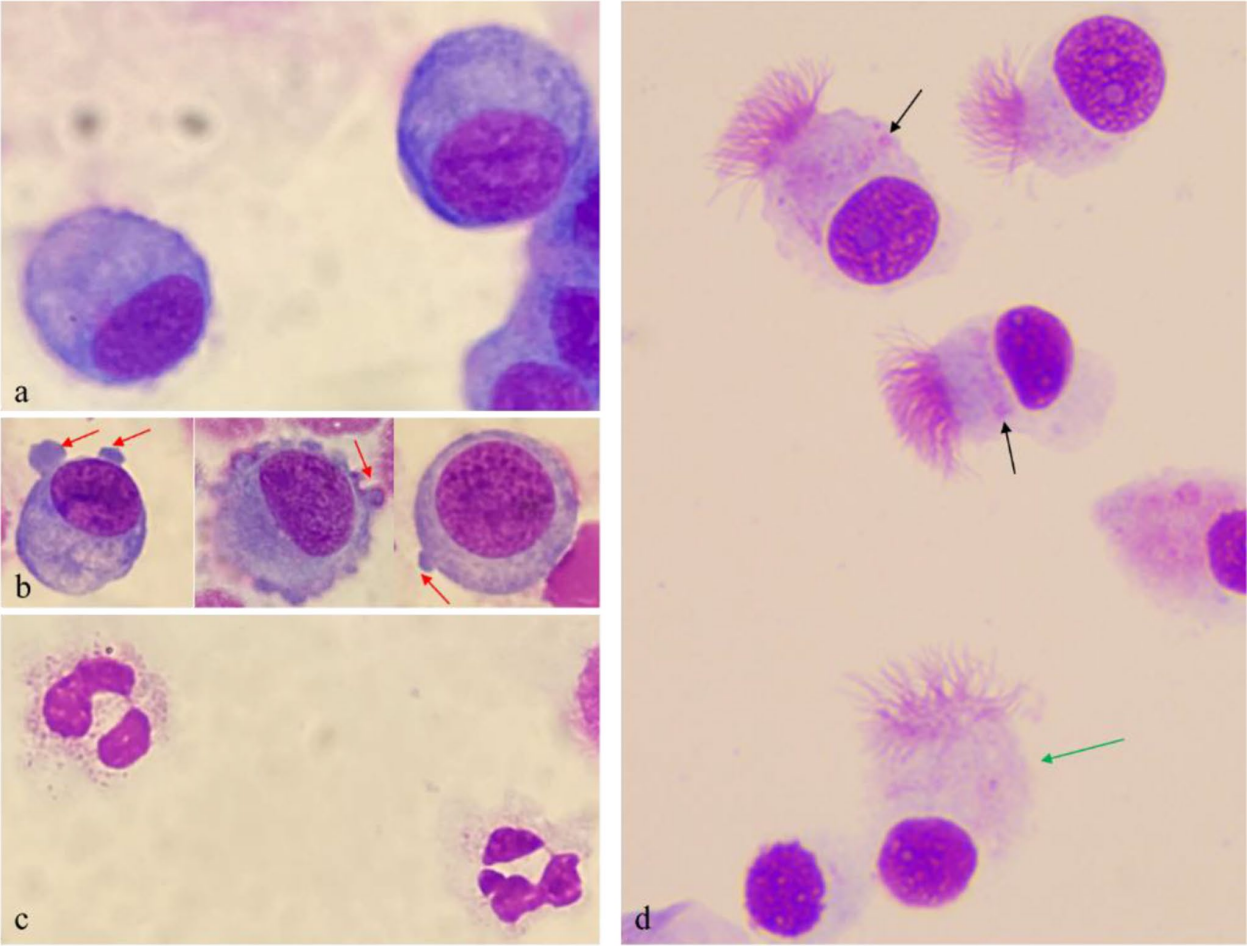
Nasal cytology in the olfactory dysfunction (OD) group, stained with May–Grunwald–Giemsa (MGG) at ×1000 magnification. (**a**) Lymphocytes; (**b**) Lymphocytes in an active functional state with pseudopodia formation (red arrows); (**c**) Neutrophils; (**d**) Ciliated cells in various stages: some retain the hyperchromatic supranuclear stria (black arrows), while others show signs of degeneration, including loss of the supranuclear stria, rarefaction of apical cilia, initial apical decapitation, and vacuole depletion (green arrow), indicative of ciliocytophthoria

Nasal cytology analysis revealed significant cellular alterations between the olfactory dysfunction (OD) group and the two control groups (CG1 and CG2). Summary statistics are presented in Table 2; Figs. 1 and 2.

In the OD group, there was a notable increase in lymphocytes compared to both control groups. The semiquantitative grading showed that 41.7% of patients in the OD group had a moderate number of lymphocytes in large clumps (2 + grading), and 47.2% had large clumps not covering the field (3 + grading). The median grading for lymphocytes in the OD group was 2+ (IQR: 2–3), compared to 0 (IQR: 0–1) in CG1 and 0 (IQR: 0–0.3) in CG2. The increase in lymphocytes in the OD group was statistically significant when compared to both CG1 and CG2. Additionally, there was mild statistical significance between CG1 and CG2, possibly due to a slight lymphocytic infiltrate in some patients who had recovered from COVID-19 without olfactory dysfunction.

Neutrophils were also significantly elevated, with a median grading of 1+ (IQR: 0–1) in the OD group, compared to 0 (IQR: 0–1) in CG1 and 0 (IQR: 0–0.3) in CG2. Specifically, 44.4% of the OD group had few scattered cells forming small clumps (1 + grading), and 16.7% had a moderate number of neutrophils forming large clumps (2 + grading), with a statistically significant difference (*p* < 0.05) when the OD group was compared to CG1 and CG2. Eosinophils and mast cells were rare across all groups and did not exhibit statistically significant differences.

The analysis of ciliated epithelial cells revealed notable abnormalities, particularly the presence of ciliocytophthoria (CCP), in the olfactory dysfunction (OD) group. CCP was present in 44% of the OD group but was observed in only 16% of CG1 and was absent in CG2.

A correlation analysis was performed to evaluate the relationship between cytological findings and olfactory function, measured by TDI scores. Spearman’s rank correlation test revealed no significant association between the grading of lymphocytes or neutrophils and TDI scores in any group. Patients in the OD group had uniformly impaired TDI scores (mean: 17.5; range: 4–26), reflecting either hyposmia or anosmia. Additionally, the narrow range of cytological gradings, with most patients exhibiting lymphocyte gradings of 2 + or 3+, likely contributed to the difficulty in identifying a significant correlation between cellular alterations and olfactory function.

**Table 2:** Semiquantitative nasal cytology grading across study groups. Results of nasal cytology assessments across the olfactory dysfunction (OD), control group 1 (CG1), and control group 2 (CG2) study groups, utilizing semiquantitative grading methods. Analyses were conducted on at least 50 fields at 1000x magnification. grading was performed according to the semiquantitative methods described by meltzer et al. (*) [39, 40] and the rhinocytogram count method proposed by gelardi (**) [6]. The table displays the percentage and number of patients within each grading category for various cell types, including epithelial ciliated cells, neutrophils, eosinophils, lymphocytes, and mast cells

	Semiquantitative Analysis			Study groups		
	Description*	Cell count**	Grading	OD	CG1	CG2
Epithelial ciliated cells	Normal	-	N	66.0% (20/36)	84.0% (15/18)	0.0% (0/18)
	Abnormal	-	A (CCP/MN)	44.0% (16/36)	16.0% (3/18)	0.0% (0/18)
Neutrophils	None	0	0	38.9% (14/36)	72.2% (13/18)	77.8% (14/18)
	Few scattered cells, small clumps	1–20	1+	44.4% (16/36)	27.8% (5/18)	22.2% (4/18)
	Moderate number, large clumps	21–40	2+	16.7% (6/36)	0.0% (0/18)	0% (0/18)
	Large clumps not covering the field	41–100	3+	0.0% (0/36)	0.0% (0/18)	0% (0/18)
	Clumps covering entire field	> 100	4+	0.0% (0/36)	0.0% (0/18)	0% (0/18)
Eosinophils	None	0	0	83.3% (30/36)	94.4% (17/18)	83.3% (15/18)
	Few scattered cells, small clumps	1–5	1+	16.7% (6/36)	5.6% (1/18)	16.7% (3/18)
	Moderate number, large clumps	6–10	2+	0.0% (0/36)	0.0% (0/18)	0.0% (0/18)
	Large clumps not covering the field	11–30	3+	0.0% (0/36)	0.0% (0/18)	0.0% (0/18)
	Up to 25 per ×100 field	> 30	4+	0.0% (0/36)	0.0% (0/18)	0.0% (0/18)
Lymphocytes	None	0	0	0.0% (0/36)	55.6% (10/18)	77.8% (14/18)
	Few scattered cells, small clumps	1–5	1+	11.1% (4/36)	44.4% (8/18)	22.2% (4/18)
	Moderate number, large clumps	6–10	2+	41.7% (15/36)	0.0% (0/18)	0.0% (0/18)
	Large clumps not covering the field	11–30	3+	47.2% (17/36)	0.0% (0/18)	0.0% (0/18)
	Up to 25 per ×100 field	> 30	4+	0.0% (0/36)	0.0% (0/18)	0.0% (0/18)
Mast cells	None	0	0	91.7% (33/36)	88.9% (16/18)	94.4% (17/18)
	Few scattered cells, small clumps	1–5	1+	8.3% (3/36)	11.1% (2/18)	5.6% (1/18)
	Moderate number, large clumps	6–10	2+	0.0% (0/36)	0.0% (0/18)	0.0% (0/18)
	Large clumps not covering the field	11–30	3+	0.0% (0/36)	0.0% (0/18)	0.0% (0/18)
	Up to 25 per ×100 field	> 30	4+	0.0% (0/36)	0.0% (0/18)	0.0% (0/18)

## Discussion

Nasal cytology has long provided valuable insights into the cellular dynamics of nasal inflammation and immune responses, making it an indispensable tool for assessing nasal diseases [38]. Despite its utility, its application to olfactory dysfunction (OD), especially in post-viral contexts such as long COVID, remains underexplored. The olfactory cleft plays a key role in olfactory disorders, making it an important target for localized assessments [41, 42]. In this study, olfactory cleft brushing was used as a minimally invasive method to sample the olfactory mucosa, enabling detailed cytological analysis to gain insights into immune responses and epithelial changes associated with persistent post-viral OD.

Our findings revealed significant cellular alterations in the olfactory dysfunction (OD) group, including marked increases in lymphocytes - particularly type II lymphocytes-, neutrophils, and evidence of ciliocytophthoria (CCP). These findings suggest a sustained immune activation and ongoing epithelial damage within the nasal and olfactory mucosa, potentially contributing to the persistence of olfactory dysfunction.

The observed lymphocytic infiltration, notably more pronounced and unexpected than the neutrophilic increase, underscores a state of localized immune dysregulation in the olfactory cleft. Type II lymphocytes, characterized by increased euchromatin content, prominent nucleoli, and vacuolated cytoplasm, are indicative of active immunoglobulin production [43]. This aligns with findings that immune responses may remain active well beyond the acute phase of SARS-CoV-2 infection [24, 26]. Moreover, the involvement of nasal-associated lymphoid tissue (NALT) in this process cannot be overlooked. NALT, a key component of the mucosal immune system, orchestrates immune surveillance and response within the nasal cavity, promoting both local and systemic immunity [44–46]. The observed lymphocytic activity may reflect antigenic stimulation or dysregulation associated with viral infections, emphasizing the role of the nasal barrier in immunity [47, 48]. These findings offer a framework for understanding the prolonged inflammatory processes underlying OD.

Neutrophilic infiltration and CCP further underscore the ongoing epithelial damage and chronic inflammation. CCP, characterized by rarefaction of cilia, loss of the hyperchromatic supranuclear stria, condensation of nuclear chromatin, and decapitation of the apical portion of ciliated cells, reflects persistent epithelial injury [49, 50]. The elevated prevalence of CCP in the OD group compared to controls underscores the role of chronic local inflammation in impeding mucosal recovery.

Despite these cellular abnormalities, no significant correlation was found between cytological grading and TDI scores, as assessed by Spearman’s rank correlation test. This lack of association could be attributed to the narrow variability in TDI scores, with most OD patients presenting uniformly low values, and the clustering of lymphocyte gradings at 2 + or 3+. These findings suggest that while cytological abnormalities reflect ongoing inflammation, they may not directly predict the severity of olfactory impairment.

Our study builds on prior work examining the interplay between immune dysregulation and olfactory dysfunction.

Prior findings identified anti-endothelial cell antibodies (AECA) in patients with persistent olfactory impairment, suggesting a localized autoimmune response involving vascular inflammation and cytokine release [51]. Additionally, elevated levels of nasal circulating calprotectin (cCLP) were observed in post-viral OD patients, supporting the role of sustained inflammation in the olfactory cleft [52]. These findings align with our current observations, further implicating localized immune activation and epithelial damage as key factors in the pathogenesis of persistent OD.

Persistent post-viral olfactory dysfunction poses a significant challenge, particularly given the absence of standardized treatment protocols. Current guidelines recommend olfactory training as a cornerstone of management, with conflicting evidence on the use of corticosteroids [53]. Short-term oral corticosteroids, though commonly prescribed, may not allow sufficient time for neuroregeneration and could be less effective in addressing chronic inflammation [54].

Our findings suggest that the inflammation observed in the olfactory cleft, characterized by lymphocytic and neutrophilic activity, could potentially respond to long-term local corticosteroids. Prolonged topical steroid administration may help modulate local inflammation, facilitating the regenerative processes of the neuroepithelium, which typically require months to years. Nasal cytology could serve as a valuable tool to identify patients likely to benefit from such treatments, enabling a more tailored approach to managing post-viral olfactory dysfunction.

## Conclusions

This study highlights the utility of olfactory cleft brushing as a minimally invasive method for assessing localized immune and epithelial changes in post-viral OD. The observed lymphocytic infiltration, neutrophilic activity, and CCP underscore the importance of targeting local inflammation in therapeutic strategies. Incorporating nasal cytology into clinical practice could enable personalized management approaches, optimizing outcomes for patients with persistent OD. Future studies should validate these findings in larger cohorts and explore their applicability to other post-viral olfactory impairments, ultimately enhancing our understanding of immune-mediated sensory deficits.

## Data Availability

The data that support the findings of this study are available from the corresponding author upon request.
